# Flexible bronchoscopy in diagnosis and management of dual tracheoesophageal fistula: A case series

**DOI:** 10.1002/ccr3.2978

**Published:** 2020-06-16

**Authors:** Matthew D. Wong, Leanne M. Gauld, I. Brent Masters

**Affiliations:** ^1^ Department of Pediatric Respiratory and Sleep Medicine Queensland Children’s Hospital South Brisbane Qld Australia; ^2^ School of Medicine University of Queensland Brisbane Qld Australia

**Keywords:** double fistula, flexible bronchoscopy, guide wire cannulation, H‐type fistula, recurrent fistula, tracheoesophageal fistula

## Abstract

Dual and H‐type tracheoesophageal fistulae can present major diagnostic and management difficulties. A methodological approach with flexible bronchoscopy and a guide wire cannulation technique was used to diagnose, localize, and aid operative surgical management in five children with dual and H‐type tracheoesophageal fistulae. All children had successful outcomes.

## Graphical abstract

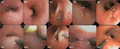

## 1 INTRODUCTION

Congenital tracheoesophageal fistulae (TEF) are bronchopulmonary foregut malformations with an incidence of 1 in 3000‐5000 live births, where 4% of cases are H‐type (H‐TEF) as described by Gross’ classification.^1^ TEF recurrence is reported in 6%‐10%^2^ and multiple TEF in 0.7%‐7%,^3^ but the true incidence of dual or multiple TEF with extrathoracic fistula is unknown.

Tracheal visualization of the fistula using rigid bronchoscopy has been favored historically to confirm the level of fistula and guide a cervical or thoracic surgical approach.^1^, ^4^, ^5^ Compared to flexible bronchoscopy, rigid bronchoscopy is technically more demanding, often distorts the airway anatomy, and may overlook a proximal extrathoracic H‐TEF. This report details how flexible bronchoscopy can be used to identify, localize, and aid surgical repair of dual TEF and extrathoracic H‐TEF.

## 2 CASES

Five patients at the Queensland Children's Hospital between 2007 and 2019 had TEF diagnosed and localized using an interventional flexible bronchoscopy technique described below. A summary of the cases is provided in Table 1. Patients A and C had dual TEF with delayed diagnosis of a second extrathoracic TEF. Patient B had an isolated extrathoracic H‐TEF. Patient D had dual H‐TEF with the second extrathoracic H‐TEF found 7.2 months after initial surgery. Patient E was diagnosed with a second intrathoracic TEF above the original fistula 1 year later.

**Table 1:** Patients with Tracheoesophageal Fistulae Diagnosed by Flexible Bronchoscopy

Patient	A	B	C	D	E
Sex	Male	Male	Female	Female	Female
Age at Diagnosis (years)	0.6	2.2	0.6	1.3	2.6 (2nd TEF) 4.2 (recurrent 1st TEF)
Co‐Morbidities	Repaired long gap EA and intrathoracic TEF, TM, tracheal bronchus	TM, BM, right vocal cord palsy, BE	Prematurity (27/40), CNLD, repaired long gap EA and intrathoracic TEF, LM, TM and aortopexy	Prematurity (32/40), grade 1 laryngeal cleft, repaired intrathoracic TEF, tracheal bronchus, BE	Prematurity (30/40), triplet, repaired EA and intrathoracic TEF, LLL BE
Presenting Symptoms					
Feed related^a^	Yes	Yes	Yes	Yes	Yes
Chronic chest rattle	Yes	Yes	Yes	Yes	Yes
Recurrent LRTI	Yes	Yes	Yes	Yes	Yes
Abdominal distention	No	No	Yes	No	No
FTT/malnutrition	No	No	Yes	Yes	Yes
Negative Diagnostic Investigations	Three AFS, one tube esophagogram, one FB	One VFSS, one AF swallow	Two AFS, two FB (one through an ETT)	None	AFS (2nd TEF) AFS, VFSS, two tube esophagograms, cHRCT (recurrent 1st TEF)
Positive Diagnostic Investigations	FB	FB	FB	FB	cHRCT, prone tube esophagogram, and confirmed by FB (2nd TEF) FB (recurrent 1st TEF)
TEF Classification	Dual TEF (Gross D), delayed diagnosis of extrathoracic TEF	Extrathoracic H‐TEF (Gross E)	Dual TEF (Gross D), delayed diagnosis of extrathoracic TEF	Dual fistula (Gross D), delayed diagnosis of extrathoracic TEF	Dual intrathoracic TEF (Gross C) including 2nd TEF and recurrent 1st TEF
TEF Repair Approach	Cervical	Cervical	Cervical	Cervical	Thoracotomy for both
Time from Diagnosis to Repair (days)	7	18	90	51	17 (2nd TEF) 138 (recurrent 1st TEF)
Postoperative Complications	Malacic spells requiring temporary NIV	None	None	Right vocal cord palsy	Pneumothorax, pleural effusion, sepsis, anastomotic leak

Abbreviations: AFS, upper gastrointestinal contrast swallow study; BE, bronchiectasis; BM, bronchomalacia; cHRCT, chest high resolution computed tomography scan; CNLD, chronic neonatal lung disease; EA, esophageal atresia; ETT, endotracheal tube; FB, flexible bronchoscopy; FTT, Failure to thrive (weight < 3rd percentile); H‐TEF, H‐type tracheoesophageal fistula; LLL, left lower lobe; LM, laryngomalacia; LRTI, lower respiratory tract infections; Malnutrition, body mass index *Z*‐score < −1; NIV, noninvasive ventilation; TEF, tracheoesophageal fistula; TM, tracheomalacia; VFSS, video fluoroscopic swallow study.

^a^ Includes choking, coughing or cyanosis.

## 3 INTERVENTIONAL FLEXIBLE BRONCHOSCOPY TECHNIQUE

### 3.1 Guide wire cannulation

**Figure 1:**
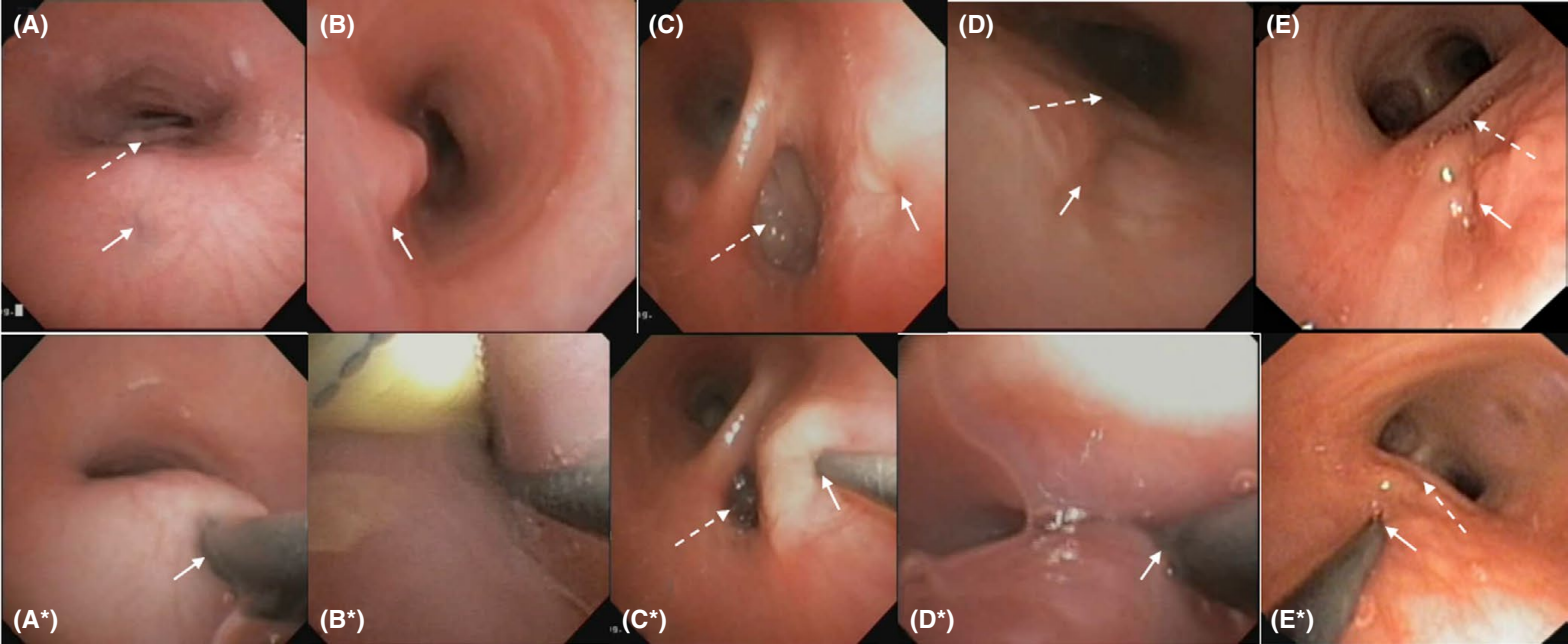
The variable appearance of dual tracheoesophageal fistulae (A, C, D, E) and a single H‐type fistula (B) displayed on flexible bronchoscopy as a subtle mucosal dimple, mucosal discoloration, an area of mucosal prominence, or within a mucosal groove (solid arrows). Four patients had previous intrathoracic fistulae repaired (A, C, D, E) and the residual pouch explored for fistula recurrence (dashed arrows). Three patients had dual fistulae with a second extrathoracic fistula found on flexible bronchoscopy (A*, C*, D*). One had dual intrathoracic fistulae (E) with the second fistula diagnosed and localized with flexible bronchoscopy (E*). Guide wire fistula cannulation was performed from the tracheal side under direct visualization with flexible bronchoscopy (A*, C*, D*, E*). One patient had a fistula large enough to pass a flexible bronchoscope through the fistula to visualize the guide wire running alongside a nasogastric tube in the esophagus (B^*^)

All procedures were performed with an anesthetist and general anesthetic (oxygen, sevoflurane, propofol, and alfentanil) in an operating room. Lignocaine 1% was applied above and below the vocal cords using a Cass needle under direct laryngoscopy. Using a nasal approach, face mask, and double swivel elbow adaptor with entry port (PH71522/S, Parker Healthcare Pty Ltd), the trachea was examined with a 3.1 mm flexible video bronchoscope (BF‐XP190, Olympus^®^ America). With the fistula in view (Figure 1), a straight or angled 0.46 mm diameter (0.018 inch) 150 cm long polytetrafluoroethylene (PTFE) coated guide wire (RF*GA18153M, RADIFOCUS^®^ Terumo Corporation) was inserted through the bronchoscope suction port to carefully probe and cannulate the fistula (Figure 1) without feeling resistance (Video S1 0:00). The wire was advanced 20 cm into the esophagus or stomach to allow enough length for ultimate retrieval. The bronchoscope was withdrawn while simultaneously advancing the guide wire to maintain a neutral wire position (Video S1 0:35). As the bronchoscope exits the upper airway (Video S1 1:00‐1:25), the wire was grasped with artery forceps at the nares and secured with tape to the patient's face, permitting removal of the bronchoscope (Video S1 1:26).

### 3.2 Guide wire retrieval

With the airway secured by orotracheal intubation alongside the wire, a bronchoscope was inserted into the esophagus or stomach to visualize (Video S1 1:54), grasp and retrieve the distal wire end (Video S1 2:15) using reusable biopsy forceps (FB‐56D‐1, Olympus^®^ America). The bronchoscope was then carefully withdrawn into the pyriform fossa (Video S1 2:26) where both wire ends were retrieved via direct laryngoscopy and Magill curved forceps, such that they now are positioned to exit through the mouth.

### 3.3 Ureteric catheter placement

A 3.0 or 4.0 French open‐end ureteric catheter (Cook^®^ Medical,) was primed with sterile 0.9% sodium chloride to reduce friction before passing it over the guide wire and through the TEF. The catheter length must measure shorter than the 150 cm guide wire but long enough to allow exposure of both ends at the mouth. With catheter ends held in the mouth, the wire was carefully removed leaving the soft catheter in situ, grasped with artery forceps, and taped to the cheek of the patient. One assistant was required to hold the guide wire, a second to advance the catheter and a third to secure the catheter after wire removal. The intraoperative use of a colored catheter bridging the TEF facilitated surgical identification within the tracheoesophageal groove intraoperatively. The catheter was manipulated by an anesthetic colleague to facilitate TEF identification by movement. It was also used to elevate the fistula higher into the neck from where it often sits behind the manubrium or clavicle. Finally, the ureteric catheter was cut and removed during fistula ligation.

## 4 DISCUSSION

In this case series, all patients had persistent complex symptoms and flexible bronchoscopy was pivotal in the diagnosis, localization, and repair of the fistulae (Table 1). While these techniques have been alluded to in rigid bronchoscopy work,^2^, ^4^, ^5^ this is the first detailed description of the procedure with an ultrathin flexible bronchoscope.

Flexible bronchoscopy has many advantages over rigid bronchoscopy including accurate visualization of subtle mucosal abnormalities of H‐TEF (Figure 1), precise localization of the level of fistula to aid surgical planning,^1^, ^5^ and minimization of airway trauma. Guide wire probing, passage, and retrieval under direct vision aid the intraoperative process while permitting excellent airway control. The passage of a ureteric catheter over the wire should be done with care as it passes alongside a noncuffed endotracheal tube. While the catheter step could be omitted, it assists intraoperative TEF identification through the addition of color into the surgical field and, compared to a guide wire, can be safely cut during fistula ligation while reducing risk of laryngeal trauma when removed.

Diagnosis of pediatric dual TEF and H‐TEF has no standardized endoscopic approach.^1^, ^2^, ^4^, ^5^ The cases presented were challenging with diagnostic delays and falsely negative radiological investigations (Table 1) before being identified with flexible bronchoscopy (Video S1). In this case series, contrast swallow studies and tube esophagograms did not provide detailed localization and often missed the TEF.^2^, ^5^ Accurate localization of extrathoracic TEF allows for surgical planning of a cervical approach, which carries less risk than a thoracotomy. Fistulae as low as the manubrium may be gently pulled to an extrathoracic position using the catheter and approached from the neck.^5^ Intraoperative chest fluoroscopy can confirm the thoracic level of wire loop and fistula but is not essential.^4^ In patient E (Table 1), fluoroscopy confirmed the presence of dual intrathoracic TEF and a thoracic approach was necessary for the second surgical repair.

Limitations of this technique include missing subtle mucosal changes at the fistula site by an unsuspecting bronchoscopist (Figure 1). Guide wire cannulation requires considerable procedural dexterity, experience, and careful planning with respect to the anesthesia, equipment, and procedure. Once intubated, retrieval of the wire from the esophagus or stomach can be done with the pediatric bronchoscope in a timely fashion. However, the small forceps size requires patience and dexterity. Without insufflation of the esophagus and stomach, the guide wire can be difficult to find using a bronchoscope (Video S1). A gastroscope and larger forceps may be helpful in this scenario.

This interventional flexible bronchoscopy and guide wire cannulation technique can aid TEF diagnosis and specifically the intraoperative management of TEF.

## CONFLICT OF INTEREST

None to declare.

## AUTHOR CONTRIBUTIONS

MDW: drafted the manuscript and contributed to study conception. LMG: contributed to the study conception and provided critical manuscript revisions. IBM: substantially contributed to the conception and methodology of the bronchoscopy technique described in this manuscript and provided critical revisions. All authors approved the final manuscript.

## ETHICAL APPROVAL

This project was approved by the Children's Health Queensland Hospital and Health Service Human Research Ethics Committee.

## Supporting information

Video S1
